# “Maintaining hope:” challenges in counseling latino patients with advanced cancer

**DOI:** 10.1097/OR9.0000000000000028

**Published:** 2020-07-28

**Authors:** Rosario Costas-Muñiz, Olga Garduño-Ortega, Normarie Torres-Blasco, Eida Castro-Figueroa, Francesca Gany

**Affiliations:** a Department of Psychiatry and Behavioral Sciences, Memorial Sloan Kettering Cancer Center, New York, NY; b Weill Cornell Medical College, New York, NY; c Department of Psychiatry and Human Behavior, Ponce Research Institute, Ponce Health Sciences University, Ponce, PR

**Keywords:** Cancer, Counseling, Depression, Health Care Disparities, Hispanic Americans, Healthcare Providers, Minority Health, Psychosocial Oncology

## Abstract

**Background:**

Latino cancer patients are at risk of poor psychological adjustment. Therapeutic effectiveness in treating Latino cancer patients with advanced cancer requires managing distress, therapeutic skill, and cultural competency. This mixed-methods study explored mental health providers’ perceptions of the challenging aspects of counseling and caring for Latino patients with advanced cancer.

**Methods:**

Mental health providers providing services to Latino or Hispanic cancer patients received an emailed web-based survey with open- and closed-ended questions. Providers included psychiatrists, psychologists, social workers, counselors, and other mental health professionals. We invited 154 providers to participate from July 2015 to January 2017. One hundred and four accessed the survey, and 66 eligible providers responded, for a response rate of 43%. Analyses were used to explore whether clinical experience factors and training characteristics were associated with perceiving conversations about cancer (diagnosis, prognosis, and end-of-life) as challenging. Second, the challenging aspects of these conversations were explored qualitatively. Four independent coders coded responses; an inductive content analysis was utilized to analyze the data.

**Results:**

Mental health providers describe encountering many challenges in their therapeutic discussions with Latino cancer patients.

**Conclusions:**

It is imperative to understand the factors associated with the perceived difficulty of these conversations, as well as the characteristics of these conversations, to develop culturally sensitive interventions and programs for patients and training interventions for providers.

## 1 Introduction

Cancer is the second leading cause of death globally and is responsible for an estimated 9.6 million deaths in 2018.^1^ Worldwide, about 1 in 6 deaths is due to cancer and approximately 70% of deaths from cancer occur in low- and middle-income countries.^1^ In Latin America and the Caribbean, prostate and breast cancers are the most common incident sites in men and women, respectively.^2^ Breast cancer is the most common cause of cancer death in Latin American women, whereas prostate and lung cancers make similar contributions to cancer mortality in Latin American men.^2^ Cancer is also the leading cause of death of Latinos in the United States, accounting for approximately 42,700 deaths/year in the US Latino population.^3^ Compared to non-Hispanic whites, Latinos are more likely to be diagnosed with advanced cancer.^3^

Two critical issues that affect the Latino population with advanced cancer are emotional distress and depression. In a meta-analysis of 94 studies with cancer patients, the authors found a reported 14% prevalence of major depressive disorder, 10% prevalence of anxiety, and a 29% prevalence of any mood complication.^4^ Another systematic review with 66 studies found that estimated prevalences of depression in different groups were: 5% to 16% in outpatients, 4% to 14% in inpatients, 4% to 11% in mixed outpatient and inpatient samples, and 7% to 49% in palliative care.^5^ This is particularly salient as a diagnosis of depression is a predictor of poor survival.^6^

In a systematic review with ethnic minority patients with cancer, Latino cancer patients living in the United States report higher distress and poorer quality of life than non-Latino patients,^7^,^8^ and Latinos diagnosed with cancer are less likely to access services from mental health professionals.^7^,^9^ Addressing the emotional impact of an advanced cancer diagnosis can be very challenging. Mental health providers have a crucial role in treating and addressing the depression, anxiety, emotional distress, and other existential and spiritual needs that emerge during the advanced disease phase.^10^

Prognosis and end-of-life conversations can be quite challenging for Latino patients with advanced cancer, their family members, and their providers.^11^ Cultural sensitivity should be a core element in the delivery and service of cancer and end-of-life care, as it affects how patients understand, experience, and respond to illness and death.^12^ Throughout treatment, mental health professionals can offer meaningful contributions to patients with advanced disease, from when an advanced diagnosis is initially shared, through treatment, palliative care, and the death and dying process.^13^

Some end-of-life studies conducted with Latino patients with advanced disease have noted unique challenges for end-of-life care in Latinos, such as denial of death or disease progression,^11^ secrecy about prognosis,^11^ and the collective, family-centered system influence on medical decision making.^11^,^14^ Within the immigrant patient population, especially the less acculturated, there is often a lack of advanced care planning and prognostic understanding, increased medical mistrust, specific religious practices, and cultural beliefs that may impact adjustment and decision making at the end of life.^15^

Research on the influence of culture in the context of advanced cancer is needed. A review of the palliative care literature on Latinos published in 2014 found that no articles had outlined which Latino cultural values are most salient in this setting or how to integrate cultural values into Latino end-of-life care.^16^ In response, the authors developed the culture-centered palliative care model. According to this model, the extent to which cultural values (*familismo*, *personalismo*, respect, trust, and worthiness) are adhered to depend on the patient and their family's level of acculturation and stage of ethnic identity.^16^ Other authors have similarly discussed the influence of Latino cultural norms and beliefs, which can be protective or risk factors, including familism (cultural value that involves individuals strong identification with and attachment to their nuclear and extended families, and strong feelings of loyalty, reciprocity, and solidarity among members of the same family^17^), fatalism, machismo, the importance of religion and spirituality, and the experience of discrimination and trustworthiness, on end-of-life decision making and care.^18^,^19^

Cultural differences in diagnosis and prognosis awareness between the United States and Latin American countries are prominent. In a study of Cuban advanced cancer patients, only 41% knew that they had cancer, but patients who knew their diagnosis reported having less anxiety compared to patients who did not know.^20^ Similarly, in a Chilean study with 49 advanced cancer patients, 20% of the sample did not know that they had a cancer diagnosis.^21^ In the United States, Latino cancer patients prefer to receive less information about their diagnosis and staging than non-Latino patients; furthermore, Latino immigrants with limited English proficiency are more likely to be unaware of their cancer stage and desire less cancer information.^22^ Often caregivers of terminally ill Latino patients report themselves having denial of the prognosis, a preference for less information, and maintaining secrecy about the prognosis. In contrast, non-Latino caregivers more often report valuing information about the details of what to expect during the patient's dying process.^11^ Many factors lead to increased barriers to diagnosis and prognosis awareness in the Latino community. Cruz-Oliver and Sanchez-Reilly^23^ identified language, religion, and family culture as 3 main barrier themes, and they identified 4 potential solution themes: cultural sensitivity training for providers, family education in Spanish, use of community leaders and community organizations, and use of the Spanish media to raise awareness of the end-of-life care concept.

This mixed-methods study seeks to identify mental health providers’ perceptions of the challenging aspects of counseling and caring for Latino patients with advanced cancer. First, we hypothesized that clinical experience factors (ie, number of years in clinical practice, number of weekly patients, weekly clinic hours) and training characteristics (ie, type of degree and number and type of cancer-related courses and seminars attended) are associated with perceived difficulty in engaging in conversations about cancer (diagnosis, prognosis, and death and dying). Second, the challenging aspects of these conversations were explored qualitatively.

## 2 Materials and Methods

### 2.1 Procedures and Participants

Data were obtained from a web-based survey, with open- and closed-ended questions, administered to a convenience sample of mental health providers practicing in the United States, Latin America, and Spain. We invited 154 providers to participate from July 2015 to July 2017; 112 accessed the survey, including participants who received the link from colleagues. Forty-six providers either did not complete the survey (n = 38) or were not eligible due to lack of Spanish-speaking ability or not actively treating Latino cancer patients (n = 8). The final sample (*N* = 66) consisted of mental health providers currently treating Latino cancer patients and offering services in Spanish, for a response rate of 43%.

Recruitment was effectuated by sending electronic invitations to providers who were listed as offering Spanish-language counseling, mental health, and/or supportive services to Latino patients on the websites of cancer centers and clinics in the United States, Puerto Rico, Spain, and Latin America and the listservs of 2 organizations, the Interamerican Society of Psychology (600 members, predominantly from Latin America) and the American Psycho-Oncology Society (>450 members, predominantly from the United States). The electronic invitations were sent by email and included a description of the study, inclusion criteria, and a link to the web-based survey. The survey was anonymous and it was not linked to the participants’ emails. Providers completed an eligibility screener, and if they were actively providing mental health services to Latino or Hispanic cancer patients in Spanish and they were professionals working in the mental health field, such as psychiatrists, psychologists, social workers, counselors, and other mental health professionals, they were invited to participate. Ethnicity of the participant was not part of the eligibility criteria. Participants were also encouraged to share the survey link with other colleagues. The process of informed consent involved participants reviewing a form with information about the study, their rights, and the potential benefits. Participants provided implied consent by completing the survey. This study was reviewed by Memorial Sloan Kettering Cancer Center's Institutional Review Board/Privacy Board and determined to be exempt research. The datasets generated and/or analyzed during the current study are not publicly available due to Memorial Sloan Kettering Cancer Center's Data Sharing Policy but are available from the corresponding author on reasonable request.

### 2.2 Measures

The survey was designed to support a program of research aiming to culturally adapt an existing psychotherapy intervention for Latino patients. As such, the survey was comprised of questions about demographic characteristics (7 questions, ie, age, sex), their clinical practice (16 questions, ie, range of years of experience), and training background (4 questions, ie, specialized training or courses), barriers of patients’ access to psychosocial services (21 questions, ie, “Lack of referrals by oncologist or cancer provider”), questions about the acceptability and feasibility of the psychotherapy intervention (29 questions, “Do you ever use these terms or concepts when treating HISPANIC cancer patients? Meaning”), and questions exploring challenging discussions in counseling cancer patients (3 closed- and 3 open-ended questions). The complete survey took between 20 and 30 minutes to complete on average. The survey was available in Spanish and English.

This report focuses on exploring the providers’ perceptions of the challenging aspects of counseling and caring for Latino patients with advanced cancer. These questions assessed if they perceived difficulty engaging in conversations about cancer diagnosis, prognosis, and death and dying (ie, Do you find it difficult talking about their cancer diagnosis with Hispanic cancer patients?). Also, the survey included open-ended questions exploring providers’ opinions about why these conversations were challenging for them (ie, If yes, please explain why). The themes of communication about diagnosis, prognosis, and death and dying were chosen because the cancer communication literature and several studies and systematic reviews have shown that communication about the diagnosis,^24^,^25^ prognosis,^26^ and death and dying (end-of-life)^27^ are core topics in counseling cancer patients and are also critically influenced by cultural context.

### 2.3 Analyses

Descriptive statistics were conducted to examine participants’ responses, including demographics, academic, and clinical practice background questions. Chi-squared statistics were used to compare responses from providers who practice in the United States to those of providers practicing in Latin America or Spain to determine whether there were differences in their demographic, academic, and clinical practice backgrounds. *χ* ^2^ analyses were also conducted to examine whether the demographics (sex, race, practice location), educational background (academic degree and years of clinical practice), or clinical load (weekly number of cancer patients and weekly number of Latino cancer patients seen) characteristics were associated with finding it difficult to talk about cancer diagnosis, prognosis, and death and dying. Qualitative analyses of open-ended responses were completed by 4 independent coders utilizing thematic content analysis to analyze the data. Interviews were coded using an open coding approach; transcribed interviews were coded by marking passages of text with phrases indicating content of the discussions. Using the report and query functions of ATLAS.ti, the qualitative analysts independently coded the transcripts and then discussed points of divergence and convergence. These discussions continued until the group reached consensus on the code meaning and application. Intercoder reliability was conducted through team-based consensus building. All the investigators had expertise in qualitative analysis and the first author moderated these discussions.

## 3 Results

A total of 66 providers of mental health and counseling services for Latinos diagnosed with cancer participated in this study. Half practiced in Latin American countries (of those approximately 40% in South America, 30% in the Caribbean, and 27% in Central America) and Spain, and the other half practiced in the United States. All participants offered services in Spanish. Most of the sample identified their ethnicity as Latino or Hispanic (89%) and two-thirds (62%) identified their race as White. Almost half of the sample had a Master's degree (49%), 38% had been in clinical practice for ≥11 years, 41% treated between 11 and 30 cancer patients per week, and almost half (46%) treated between 6 and 20 Latino cancer patients per week (Table 1).

**Table 1:**
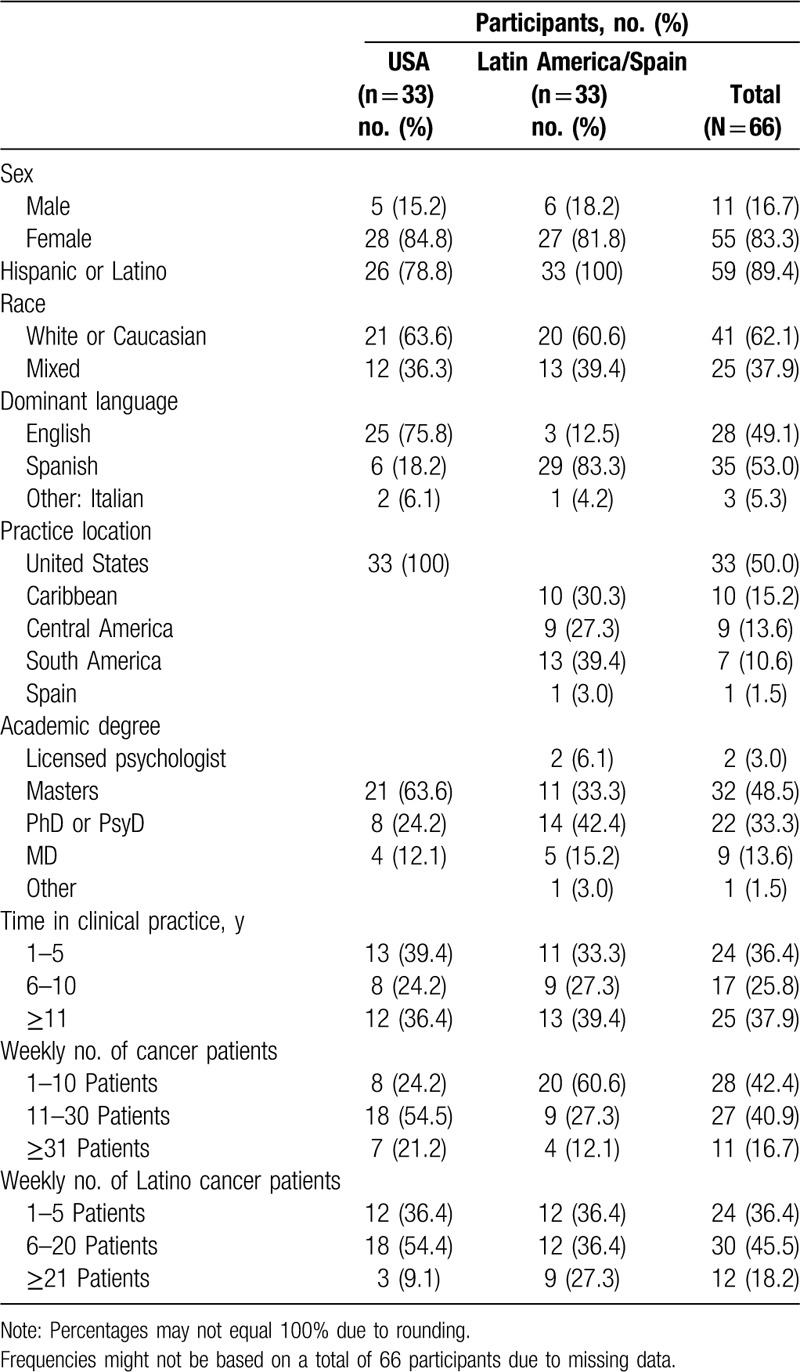
Characteristics of mental health providers by region.

Nine percent of providers reported that they found it difficult to talk with their Latino cancer patients about their cancer diagnosis. One in five mental health providers (20%) found it difficult to talk about the patient's cancer prognosis, and one-quarter (26%) found it difficult to talk about death and dying. Thematic categories highlighting challenging aspects of discussion across the 3 areas (diagnosis, prognosis, and death and dying) were: emotional impact and coping, cultural issues, and patient-provider related themes. Relevant themes in conversations about cancer prognosis and death and dying were lack of information, cancer-related education, and/or communication about cancer.

### 3.1 Communication about Diagnosis

None of the demographic, academic background or clinical load characteristics were associated with finding it difficult to talk about a diagnosis of cancer with Latino cancer patients. However, having taken courses in health psychology, psychosomatic, or behavioral medicine (*χ* ^2^ = 17.18, *P* = .000) and attending cancer-related, psycho-oncology, or supportive oncology conference workshops/seminars (*χ* ^2^ = 8.70, *P* = .01) were associated with not finding it difficult to talk about a diagnosis of cancer (Table 2). Some of the providers who found it difficult to talk about cancer diagnoses reported that it was because some patients refused to accept the diagnosis, had limited information, or were “unclear about the diagnosis, staging, or name of their cancer.” See Table 3 for exemplary quotations. Other providers stated that they found cancer diagnosis conversations difficult because they felt that they did not have the necessary “tools” or needed more training and that these conversations are facilitated by the cultural competency of the provider and building patient-provider trust.

**Table 2:**
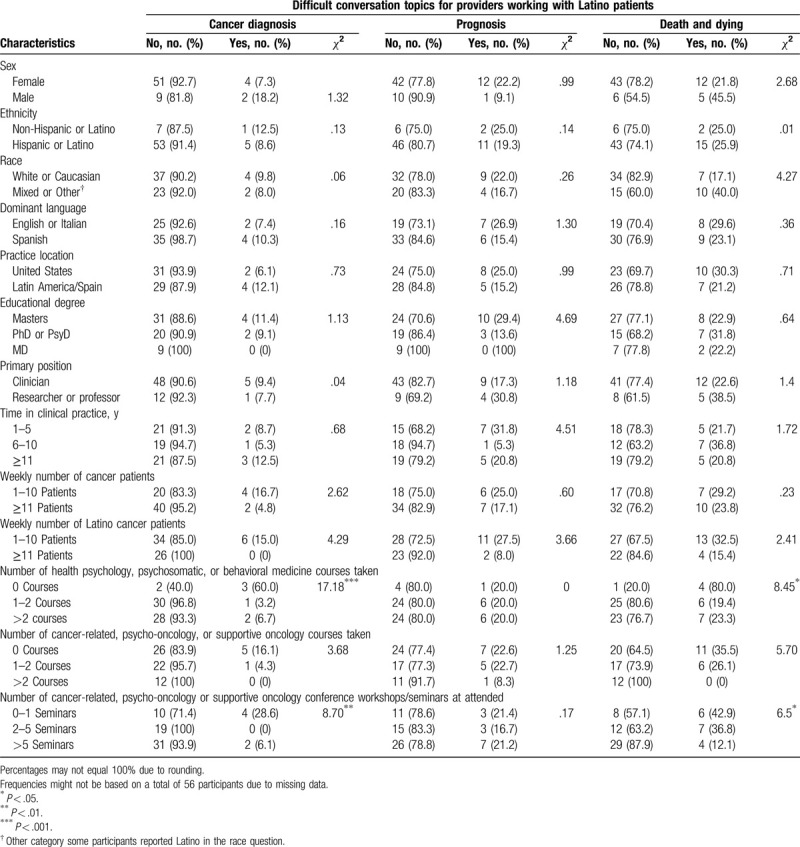
Characteristics of mental health providers and associations with perceived difficulty in discussing cancer diagnosis, prognosis, and death and dying topics.

**Table 3:**
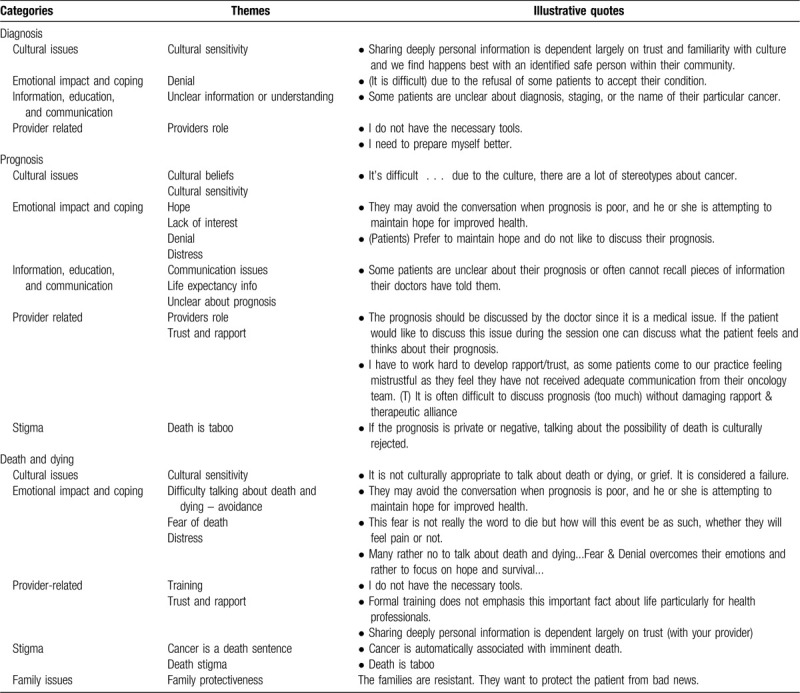
Difficult topics in conversations about diagnosis, prognosis, and death and dying—qualitative results.

### 3.2 Communication about Prognosis

None of the demographic, academic background, clinical load, or training characteristics of the participants were associated with finding it difficult to talk about medical prognosis with Latino cancer patients. Providers reported that having conversations about prognosis with their Latino patients was difficult because it was not culturally acceptable to talk about or accept death or grief. They felt that patients might avoid these conversations because they try “to maintain hope for improved health,” “are unclear about their prognosis or can’t recall pieces of information,” because “talking about a poor prognosis may damage the rapport or the therapeutic alliance,” and patients may “be mistrustful as they feel they have not received adequate communication from their oncology team.” One mental health provider emphasized that medical providers should discuss patients’ prognoses with them, and mental health providers should focus on addressing the patients’ thoughts and feelings about their prognosis if the patient raised the topic during a session (see Table 3).

### 3.3 Communication about Death and Dying

None of the demographic, academic background, and clinical load characteristics of the participants were associated with finding it difficult to talk about death and dying with Latino cancer patients. Having taken courses in health psychology, psychosomatic, or behavioral medicine (*χ* ^2^ = 8.45, *P* = .02) and attending cancer-related, psycho-oncology, or supportive oncology conference workshops/seminars (*χ* ^2^ = 6.57, *P* = .04) were associated with less difficulty in talking about death and dying (see Table 2). Providers perceived death and dying discussions as being difficult because: “It is not culturally appropriate to talk about death or dying”; patients avoid this conversation topic to “maintain hope for improved health”; patients are overcome with fear and denial and “would rather focus on hope and survival”; and that “Cancer is automatically associated with imminent death.” Providers perceived that family members might resist talking about or addressing end-of-life topics, patients might avoid talking about dying because of concern about the impact on their family, and patients often talk about concerns for their family when they die. Finally, some providers reported that they needed more training and that “formal educational training (ie, graduate school) does not include this important fact about life” (see Table 3).

## 4 Discussion

Counseling Latino patients during the cancer experience and discussing issues around diagnosis, prognosis, and end of life can be challenging for psycho-oncology providers. The process of counseling Latino cancer patients from the perspective of mental health providers has not been previously examined. The current study's findings describe that perspective and illustrate how providers who have taken courses in health psychology, psychosomatic, or behavioral medicine perceive less difficulty in engaging in conversations about cancer diagnosis and end-of-life issues with their patients. Providers perceived that cultural competency facilitates communication about diagnosis, prognosis, and end-of-life conversations, but many reported needing additional training on how to address these topics with patients. They also expressed that a main barrier to having conversations about prognosis and death and dying was that it was not culturally appropriate to talk about death and that patients perceived conversations about prognosis and end of life conflicted with maintaining hope for survival and improved health. Other challenging aspects of having these conversations were a lack of accurate information or poor comprehension about prognosis among patients, which may have engendered a sense of medical mistrust related to feeling that the communication they had received from their cancer care team had been inadequate. Finally, patients might avoid conversations about diagnosis, prognosis, and death and dying due to emotional distress and concerns associated with their family members’ well-being and reactions.

Providers reported that talking about the cancer diagnosis was facilitated by the cultural competency of the provider, perceiving that “sharing deeply personal information is dependent largely on trust and familiarity with the culture.” Understanding cultural values and the context of different ethnocultural groups is vital for effective and sensitive counseling.^28^,^29^ In the context of palliative care, several studies have shown the importance of cultural sensitivity, given the intrinsic role of culture in patients’ and their families’ adaptation.^19^,^30^ Culture impacts how patients experience their health conditions, respond to symptoms, make health care decisions, and express distress related to the dying process.^31^ Therefore, it is imperative for health care providers of Latino cancer patients to be attentive to how the cultural background might influence adaptation and decision-making regarding cancer care. This is particularly important when the provider does not share the same cultural background as their patient, which is often the case in United States. For example, in this sample, 11% of the providers were not of Latino, Hispanic, or Spanish decent. Nonetheless, regardless of ethnicity, although clinicians should be attuned to the culture and needs of patients, they should refrain from stereotyping, and assuming that cultural scripts apply to all Latino patients.

Providers in this study reported that having prognosis conversations with Latino patients was difficult because of these reasons: some patients refuse to accept their diagnosis; it is not acceptable to talk about death or grief in Latino culture; and talking about a poor prognosis may damage the patient-provider rapport or the therapeutic alliance. Some patients may avoid these conversations “because they try to maintain hope for improved health,” their fear and denial overcome their emotions, and they would rather focus on hope and survival. In a systematic review looking at sustaining hope in palliative care patients, some studies reported that a minority of patients and caregivers avoid detailed information to preserve hope.^32^ They found that a minority of health care providers also avoid giving information to promote hope.^32^ However, other studies have shown that the provision of practical information in a supportive, collaborative environment could instill more hope than avoidant behavior.^32^ Overall, the literature supports the importance of respecting patients’ and caregivers’ individual preferences. Providing undesired information or engaging in prognostic conversations in an untimely and unsuitable manner does not respect individual autonomy because patients have the right to refuse this information. The best approach may be to encourage patients to have conversations on their terms and ascertain what kinds of discussions they want to have or to avoid (ie, talk about logistics and plans; talk about fears, emotions, and coping; talk about the impact on the family; and/or engage in existential reflections). Additionally, providers should respect patients’ preferences and corroborate at multiple time points, rather than addressing them in one-time conversations, since the patient's needs and desires can change over time.^32^

Providers reported difficulty in engaging in conversations about cancer because some patients have limited information or are unclear about diagnosis, staging, prognosis, and/or the name of their cancer, or cannot recall pieces of information, and patients may have become mistrustful of providers, feeling that they have not received adequate communication from their oncology team. In a study conducted with Latino cancer patients, two-thirds of the sample (65%) had no knowledge of their cancer stage, 38% were unaware of the metastatic state of their tumor, and only 15% of patients expressed that they would like additional information about their diagnosis and/or treatment.^22^ Having limited information about cancer might be a consequence of language barriers, limited health literacy, poor communication/trust in providers, a passive information-seeking approach, and/or information avoidance to maintain hope in the face of disease decline.

Health literacy is defined as “the degree to which individuals have the capacity to obtain, process, and understand basic health information and services needed to make appropriate health decisions.”^33^ Limited health literacy is prevalent among patients in the United States (46%) and even more prevalent among Spanish-speaking patients (62%).^34^ Poor communication outcomes are also more prevalent in Latino patients, mainly due to language barriers.^35^ Patients with limited health literacy more often use a passive communication style with their providers, do not engage in shared decision-making, and report that interactions with their physicians are not helpful or empowering.^36^,^37^,^38^,^39^

Providers perceived that family members might resist talking about or addressing end-of-life topics, resulting in patients avoiding talking about dying due to concern over its impact on the family, although patients often talk about concerns for their family after they die. Family members of Latino patients might have a preference for secrecy, denial as well as limited patient autonomy and exchange of information, especially when the patient is dying, attempting to shield the patient from information they believe might be harmful to them.^11^,^40^ Gaudio et al^19^ conducted a qualitative analysis of Latino cultural factors in the context of palliative care, finding that the emphasis on close relationships seen in Latino families can be helpful when confronting a terminal diagnosis because some large families can draw on ample support and resources. However, these close relationships can be harmful when family members have competing agendas or when there is family fragmentation.

Encouragingly, providers in this sample who had taken courses in health psychology, psychosomatic, or behavioral medicine and those who had participated in psycho-oncology or supportive care seminars or workshops perceived less difficulty in engaging in conversations about diagnosis and end-of-life issues with their patients. Some providers reported that they found it difficult to counsel cancer patients with advanced disease because they felt that they did not have the “tools” or needed more training. This was particularly salient for some providers who reported that they needed more training in end-of-life care. Training opportunities for psychologists in palliative or supportive care are scarce. Several online trainings have been developed; however, online training can be insufficient for clinicians to feel confident about treating patients with advanced disease, if the trainings do not combine didactic and clinical practice.^41^ Formal training programs should include a mix of didactics, supervision, and hands-on clinical care of patients.^41^

The palliative care literature often cites language and religious issues as influencing and complicating the care of Latino cancer patients with advanced disease.^19^,^23^ However, this sample of mental health providers did not cite these factors as challenging. All providers in the sample were Spanish speakers; therefore, language is not a relevant barrier in their work. Furthermore, as religious coping and beliefs were not identified as posing challenges to the providers surveyed, it is possible that the providers perceived religious beliefs as playing a neutral or an effective role in coping and adjustment.

The literature on cancer care communication has focused on examining preferences and skills of oncologists. One study found that up to 84% of oncologists reported to be at least slightly burdened by having to break bad news to patients (ie, anticancer treatment not effective anymore).^42^ Further, Baile et al^43^ found that oncologists reported moderate to high levels of difficulty in discussing diagnosis, prognosis, and end-of-life issues. Not surprisingly, oncologists report high levels of difficulty and burden when having these conversations. To the best of our knowledge, this is the first study exploring the perceived difficulty by mental health providers of communicating these 3 issues with cancer patients. However, our findings are limited to assessing difficulty in these three issues. Future studies with mental health providers should include a more comprehensive assessment of communication comfort, confidence, and specific challenges.

### 4.1 Clinical Implications

The current study has illustrated several areas of need that were identified by providers regarding the psychosocial care of Latino cancer patients, such as the need for culturally sensitive services and the need for specific training opportunities. Cultural sensitivity training in the areas of palliative and end-of-life issues and care is warranted, even for those who share the same language as their patients.

In developing these types of trainings special attention needs to be given to the skills being taught. Epner and Baile^44^ caution taking a multicultural approach to cultural competence training, as it may result in stereotypical thinking rather than the development of clinical competence. They recommend a newer, cross-cultural approach to culturally competent clinical practice focusing on foundational communication skills, awareness of cross-cutting cultural and social issues, and health beliefs that are present in all cultures.^44^ The patient-centered approach should rely then on identifying and negotiating different styles of communication, decision-making preferences, roles of family, sexual and sex issues, and issues of mistrust, prejudice, and racism, among other factors.^44^ Specifically, this type of training could aim to develop and advance providers’ skills in addressing challenging situations in counseling, such as patients’ denial and lack of acceptance of their diagnosis and/or poor prognosis, cultural avoidance of death and grief, limited information about diagnosis and prognosis, limited health literacy, mistrust, and family-related resistance to open communication about cancer diagnosis and prognosis.

Furthermore, in another study with this sample of providers,^45^ they reported using these therapeutic approaches: acceptance and commitment, cognitive behavioral, problem-solving, psychodynamic, narrative, mindfulness, humanistic, meaning-centered, patient-centered, supportive, motivational interviewing, and integrative therapies; and communication strategies: empathic listening, active listening, normalizing, refraining, reflecting, validation, using hopeful language, and other therapeutic techniques included breathing exercises, behavioral interventions, decisional balance, existential strategies, here-and-now, relaxation, visual techniques, written techniques, and psycho-education when treating Latino cancer patients. However, it is unclear whether they are consistently using evidence-based interventions found effective with cancer patients, as many of these interventions have not been tested with Latino cancer patients.^45^

Providers that have taken courses in health psychology, psychosomatic, or behavioral medicine and have attended cancer-related, psycho-oncology, or supportive oncology conference workshops/seminars reported with less difficulty in talking about death and dying. More trainings are needed to increase providers’ confidence and comfort in engaging in discussions about death and dying. As stated, these trainings should include attention to culturally sensitive care, but also aim to teach skills to improve communication skills, management of patient's and family's fear and denial, and patient's unfinished businesses and family-related concerns. Finally, communication in counseling is key and core to therapeutic effectiveness and alliance. Some programs have demonstrated success in improving the communication skills of clinicians in cancer.^46^,^47^,^48^ Demonstrating sympathy and empathy, having the capacity to engender trust, finding appropriate and timely ways to talk about death and dying comfortably and without fear, and involving the patient and family in open communication are critical skills for counselors and mental health providers working with patients with advanced illnesses and their family members.

### 4.2 Study Limitations

This study had several limitations. First, the validity of the qualitative responses is limited by design because they were elicited from a survey with open-ended questions. Future studies should be conducted using in-depth interviews. However, this design facilitated a more comprehensive sample, allowing the recruitment of providers in distant geographical locations and eliciting a response rate of 43%. Second, the small sample size precluded us from conducting multivariate analyses to examine associations between background and practice characteristics and the perception of difficulty in counseling Latino patients with advanced disease. Third, this was an anonymous survey and information from nonresponders was not collected. Given this was not a random or representative sample, results cannot be generalized to providers counseling Latino cancer patients. Given these limitations, this study captures the perspectives of mental health professionals, thereby making an important contribution to the psycho-oncology field. A next step will be to conduct a larger study examining the practice-related factors and training needs of Spanish-speaking mental health providers of Latino cancer patients.

## 5 Conclusion

It is critically important to understand the factors associated with the perceived difficulty of these conversations, to develop effective interventions and programs for patients and training interventions for providers. Studying the provider perspective regarding the challenges of providing counseling to Latino cancer patients and the characteristics of these conversations provides a unique perspective on the most prominent psychosocial challenges Latino cancer patients face.

## Conflicts of interest statement

The authors declare that they have no financial conflict of interest with regard to the content of this report.
